# Above and Beyond: A Grounded Theory of Aotearoa/New Zealand High School Teachers’ Perspectives on International Study Tours

**DOI:** 10.1007/s40841-023-00291-6

**Published:** 2023-05-19

**Authors:** Donna O’Donnell, Mark Orams, Heike Schänzel

**Affiliations:** School of Hospitality and Tourism, Auckland University of Technology, 55 Wellesley Street, East Auckland City, Auckland, New Zealand

**Keywords:** Educational tourism, High schools, Teachers, Grounded theory

## Abstract

This paper addresses the dearth of research into the roles high school teachers play in organising and leading international study tours offered by high schools in New Zealand (prior to the COVID-19 pandemic). The aim of this paper is to provide insights into the motivations and experiences of teachers involved in these tours. A grounded theory approach was used, and qualitative data were collected via face-to-face interviews with eight teachers forming the basis of the development of a theory which proposes that both navigating and negotiating learning experiences are key aspects of the teacher’s role. Data revealed that the expectations and challenges placed upon the teachers had implications for their personal and professional lives. The tension between teachers’ perceived obligations for the safety of the students and the adolescent’s desire for freedom to explore whilst travelling proved difficult to resolve and teachers questioned the sacrifices they personally needed to make.

## Introduction

For over a century educational opportunities outside the classroom (EOTC) have been offered in New Zealand schools and form a prominent part of the formal school curriculum (Ministry of Education, 2018). In recent years these opportunities have expanded and diversified to include international travel experiences. Prior to the Covid-19 pandemic, a general trend was that high schools in New Zealand offered international study tours as part of the school curriculum and/or for extra-curricular learning. Disruptions to international travel such as those caused by the COVID-19 pandemic from 2020 to 2022, are not new. In the past there have been numerous disasters and crises that have imposed restrictions on travel. Historically, waves of disaster and a commensurate decline in travel have been followed by new phases of growth and prosperity for tourism. Such a pattern was evident when the New Zealand tourism industry grew by 11.5% in 2004 after SARS epidemic (Collier, 2007). It is anticipated, therefore, that overseas study tours will resume in high schools and that growth in these offerings, widespread prior to COVID-19, will recommence. This study used a grounded theory approach to provide in-depth insights into the motivations and experiences of teachers involved in organising and leading international study tours for New Zealand high-school students pre-pandemic which formed the basis of the development of the navigating and negotiating learning experiences theory.

## Literature Review

Research from the fields of education and tourism has consistently shown the connection between learning and travelling (Porter, 2018). For example, Smith and Clayton (2009) and Campbell-Price (2014) recognize that travel experiences can act as informal qualifications. Indeed, it is generally acknowledged that school leaders, teachers and students all perceive education outside the classroom to be beneficial (Pike, 2020).

The growing popularity of international tours is evident from media coverage and high school websites, with high schools in New Zealand offering an increasingly diverse range of opportunities to their students (Gerritsen, 2020; Redmond, 2017). Pre-COVID-19, further evidence of the rise of this specific educational tourism offering is provided by the growth of private companies focused on this market such as Travelbound or Defining Moments (Defining Moments, 2019; Travelbound Education, 2019). Educational tourism experiences, especially in high schools, have both expanded and diversified with opportunities ranging from subject-based curriculum derived study tours which focus on specific subjects such as languages, geography, history, classics, music, and the performing arts.

In New Zealand, government policies and agendas have contributed to the development and growth of international study tours with the Ministry of Education explicitly encouraging high schools to offer alternative pedagogies in the delivery of the curriculum to address the diverse interests and learning needs of students and respond to changing societal needs for the twenty-first century (Ministry of Education, 2018). This has coincided with growing global trends towards globalisation, referring to the increasing interconnectedness and interdependence of the world’s economies, societies, and cultures (McGrew et al., 2007), and cultural understanding influencing the New Zealand curriculum (Ministry of Education, 2018). This in turn has fostered the development of learning opportunities that incorporate overseas travel.

Although extensive research has been conducted on educational tourism, the majority of this has focused on the perceived benefits of participating in international study tours from a student perspective. Examples include research that has explored the effects of participation on the personal development of students (Penning et al., 2019), their social skills (Williams et al., 2018), cultural understanding and global citizenship (Schänzel & O’Donnell, 2021; McGladdery & Lubbe, 2017). Additionally, there is a wide range of research that examines the effect of study tours on student’s language learning and acquisition (e.g., Hiver & Sánchez Solarte, 2021); and on student’s subsequent academic and career success (Bretag & Van der Veen, 2017; Ruth et al., 2019).

In contrast, limited research exists on the teacher’s perspective with published research to date tending to focus on the teacher’s perceptions of student learning as opposed to their own experiences. For example, More’s (2011) study focused on how a visit to an historical village provided opportunities to build academic skills in students. The scant literature presenting the teacher’s perspective has generally focused on domestic fieldtrips as opposed to international study tours. Johnston (2015) for example, reviewed his domestic biology field-trip, discussing the stress associated with preparing and planning the trips. Further studies have focused on the importance of preparing and planning out-of-classroom experiences. Coll et al. (2018) and Anderson and Zhang (2003) emphasised the need for proper planning so learning opportunities fit within the curriculum. Harvey (2018) noted that out of classroom experiences can be time consuming, and teachers involved in organising and delivering such experiences may be subject to many pressures and constraints imposed by both government legislation and parent’s expectations.

While a review of relevant literature indicates that extensive research has been undertaken on international study tours generally, little research has focused on high schools or on the teacher’s perspective despite the scale and growth of these tours. The research presented in this paper seeks to address this gap by presenting insights into the teacher’s perspectives in a New Zealand high school setting and offering a theory which explains key aspects of the findings from these teacher’s lived experiences.

## Research Design and Methods

A constructivist grounded theory (CGT) approach was used to provide insights into the lived experiences and perspectives of high school teachers in New Zealand in relation to their organisation of and participation in international study tours for high school students. CGT is a a collaborative process between the researcher and participants to construct knowledge and uncover personal meanings, by attempting to make sense of, and interpret real life situations (Charmaz, 2006; Denzin & Lincoln, 1994). Ontologically and epistemologically, the researcher recognised that knowledge was socially constructed from individual experiences, subjective, and context dependent, with different individuals having different perspectives and they became an integral part of the research process, (Charmaz, 2006), acknowledging that their identity (positionality) and personal biases may influence the research process (Birks & Mills, 2015). Consequently, engaging in the process of reflexivity constantly reviewing the data, questioning their own understanding to ensure the data was representative of the participants experiences. Adopting a grounded theory approach was key to understanding the individual teacher’s experiences of organisation and participating in international study tours in this study.

### Data Collection

Secondary school teachers that had organised and participated in international study tours were identified and purposive sampling (Denzin & Lincoln, 1994) was used to invite the teachers to participate in this research. Table 1 outlines the teaching profiles of the study participants, including information on the number of years they have been organising, and participating in international study tours, the subject/s taught, and the destination of the study tour they were involved with. Additionally, information was collated on the socio-economic status of the community the school is located within (in the New Zealand context this is expressed as a decile rating, with a range of 1–10). Decile 1 schools are the schools with the highest proportion of students from low socio-economic communities, whereas decile 10 have the lowest proportion of these students (Ministry of Education, 2015). This rating is presented because literature suggests the socio-economic status of the school’s community can influence participation in international study tours (Simon & Anisworth, 2012).

**Table 1:** Participant Profiles

Participant	Pseudonym	Subject	Decile	Destination of Study Tour	Length of Time Organising Study Tours	Type
1	Andrea	Social Sciences	10	Cambodia	12 Years	Co-Ed
2	Brooke	Language(s)	10	Various Cambodia Mexico Japan	11 Years	Co-Ed
3	Claire	Geography	10	Various Hawaii Amazon	20 Years	Co-Ed
4	Debbie	Classics	5	Italy Turkey Greece	6 Years	Co-Ed
5	Edward	Classics	10	Italy Turkey Greece	4 years	Co-Ed
6	Frank	Classics History	10	Various Italy Greece Vietnam United Kingdom	11 Years	Co-Ed
7	George	Sports Maths	10	Various Kenya South Africa	15 Years	Single Sex Boys school
8	Helen	Language(s)	5	Various Noumea France Germany	17 Years	Co-Ed

Following the process outlined by Charmaz (2006) and Denzin and Lincoln (1994) semi-structured face-to-face interviews were conducted with the teachers that had organised the international study tours. After eight interviews data collection ceased when theoretical saturation was reached, revealing no new insights from the data (Charmaz, 2006). This approach is typical for grounded theory studies, with Mason (2010) suggesting that as few as five participants can be adequate, because qualitative research focuses on in-depth understanding and meaning and not the number of interviews (Morse, 2000). Ethics approval and informed consent was gained in accordance with university policy prior to the data collection.

### Data Analysis

**Fig. 1:**
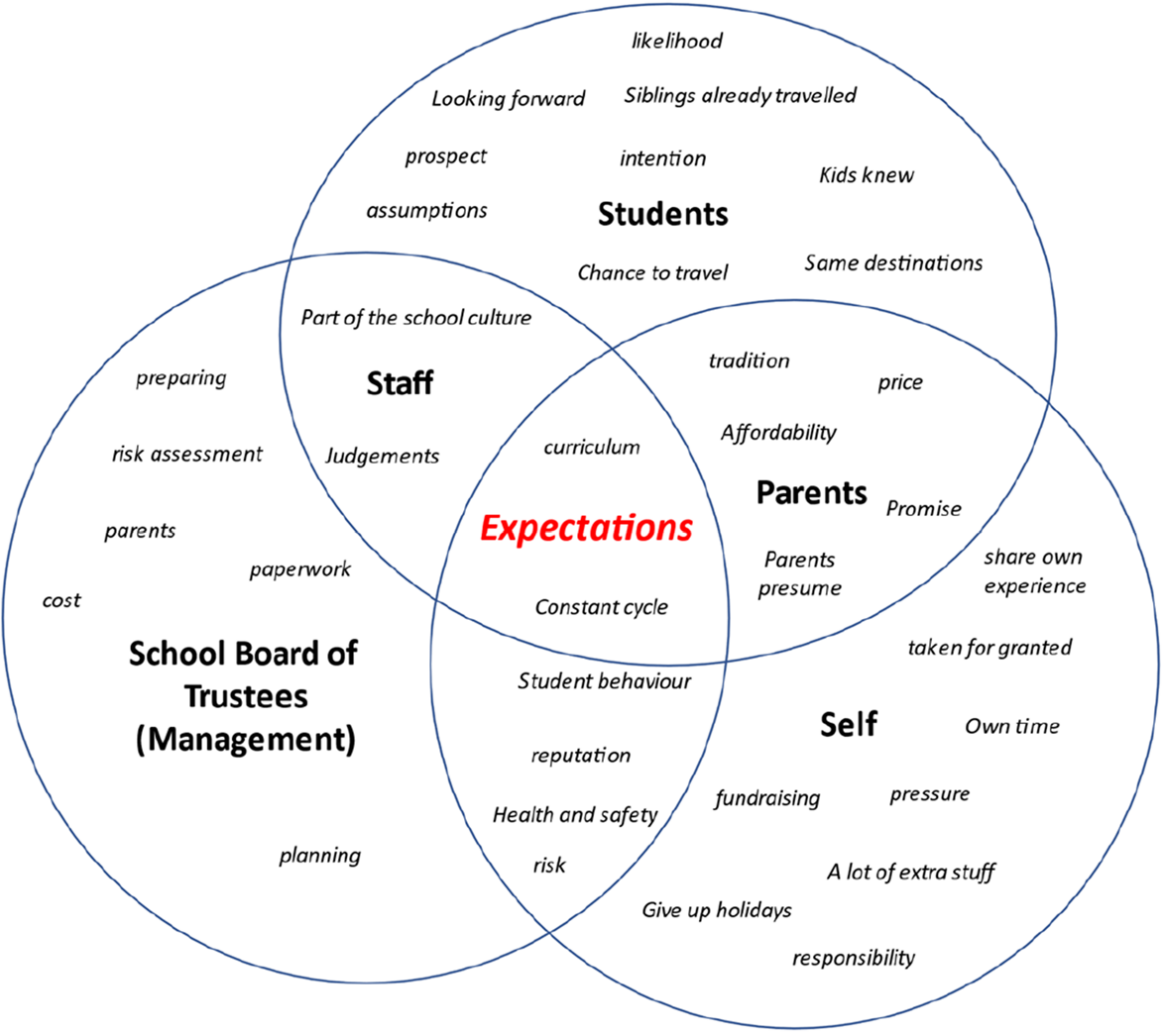
Category Construction—Expectations

Interviews were audio recorded and written transcripts were developed from the audio recordings. These transcripts formed the primary data for this study. Initially, coding was conducted manually, line by line, words were analysed for meaning and data were coded and given appropriate names. This interpretative process of examining, exploring, comparing, and reflecting allowed for similarities and differences to be identified and new categories to emerge. Subsequently, a second phase of coding (referred to as focus coding) was used to further refine the data categorization, by selecting the most frequent, and relevant codes to integrate and synthesize into larger categories (Charmaz, 2006). The cluster diagram (Fig. 1) illustrates the category *expectations* as an example to demonstrate how the category was conceptualized and dimensions incorporated. The category highlights the overlapping expectations placed on teachers and the situation they found themselves in between the students, staff, parents, and the School Board of Trustees.

**Fig. 2:**
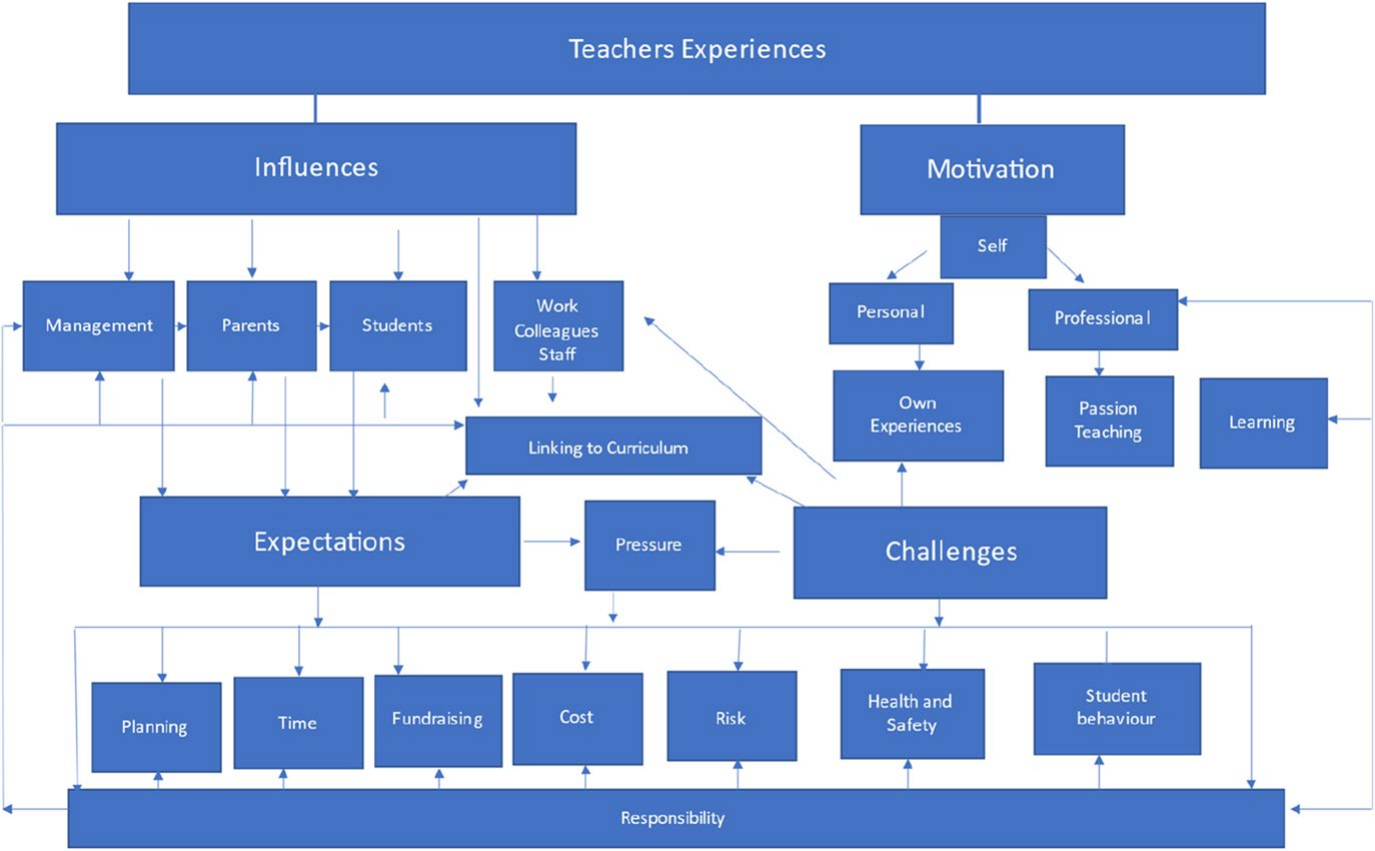
Mind map illustrating the connections in the data

The third phase of coding (referred to as axial coding) focused on making decisions to use the most predominant codes to integrate and synthesize the data and link the categories together. At this stage mind maps were utilized to enhance this process and understand the participants actions (Birks & Mills, 2015). Figure 2 shows the process of connecting the categories and developing two of the main categories (Expectations and Challenges).

The final phase, theoretical coding, is the process of merging and refining the categories using theoretical sensitivity which “involves seeing possibilities, establishing connections and asking questions” (Charmaz, 2006, p. 244). During this analysis consideration was given to the research questions for this study which focus on gaining in-depth insights into the teacher’s experiences. This four-stage analytical approach, following that outlined by Charmaz (2006) resulted in the emergence of four main themes. These themes formed the basis of the grounded theory as outlined below.

## Findings

The findings are presented according to the four main themes identified: Expectations, Navigating, Negotiating, and Challenges. These themes provide insights into the rationale and justification of the international study tours from a teacher’s perspective and focus on the influential factors motivating high school teachers to organise and conduct the study tours. Furthermore, these findings revealed the personal and professional challenges faced by the teachers to make the trips successful.

### Expectations

The data revealed that teachers had many expectations placed upon them, before departure, during the study tours and post departure. A common view was that international study tours had been embedded into the school culture and study tours had become a school tradition and, consequently, many teachers felt there was an expectation placed upon them to organise these tours. In many cases the expectations of students to participate in a study tour influenced the teacher’s decision-making process and choice of destination. For example:

It was just known that every two years the social science department would take an overseas trip, and so kids sort of knew about that (Frank).

You have to have it as an institutionalized thing, if possible, I realized it only works if it’s a regular thing; then the next generation of students know (Helen).

These teacher’s comments imply that many students were aware of the international study tours before they attended high school. Hence, incoming students to a high school expected to be able to travel to the same destinations. For many teachers this was an influential factor in the destinations selected and the organisation of the study tours. Teachers noted that over the last few years the availability and variety of study tours had expanded within the school.

There were expectations from the School’s Board of Trustees (school management/ governors) that international study tours were linked to the school curriculum for example:

An initial trip application goes to the principal—that application will then go to the board, the board will then look at this, look at your objectives (Helen).

Although on the surface the curriculum influences the organisation of the study tours, for many teachers their own personal experiences were influential on the choices made. There was a sense of passion and personal commitment to facilitate the experiences that international travel provides for adolescents. For example:

For me when I was 14 and went on an exchange, the fact that a teacher encouraged my parents to let me go, meant that my life changed forever. So, I look at it from the point of view that maybe one of these trips makes a difference like that for one, or more than one of those kids (Brooke).

Moreover, the teachers identified educational benefits within the classroom:

When we’re teaching that topic, I’ve got some students sitting in front of me that have got experience in having gone, and that is wonderful (Claire).

Comments such as these were common and indicative of the importance that the teachers placed on the international study tour opportunities. These perceived benefits provide an explanation regarding why teachers continue to engage in taking responsibility for organising and leading these tours when contrasted with the tension that revealed the personal and professional sacrifices these teachers make when undertaking these roles. Whilst the expectations and personal motivations differed from teacher to teacher, all the teachers interviewed were aligned in their views that they needed to sacrifice a great deal of their own time to ensure the international study tours were successful. These sacrifices were traded off in the teacher’s minds against the benefits created by the opportunities for the students to travel, this is evident from one teacher’s comment:

I think it’s worth it for the kids. I’m not doing this for myself. I mean, I’ve been to Rome. I’ve been to Italy. I’ve been to Greece, but for the kids—for this experience for them—to see the things that they’re seeing over there and to have that time and to be in those places I think is absolutely invaluable for them (Edward).

Such comments suggest a sense of pride and satisfaction from organising the international study tours, and a commonly held view that these experiences enhanced the learning environment for students. However, several teachers reflected on the expectations and pressures they encountered at an institutional level. While some of the teachers felt there was overall support from their school, others were less complimentary of the school management’s attitude. This is emphasised in the following comment:

They pretty quickly tapped into the positive publicity of the trip, but I would argue that in terms of the huge amount of school support, it was, yeah you can do it but you’re on your own (Andrea).

Overall, student, parent, and institutional expectations all play a role in teachers’ organisation of international study tours. This requires teachers to balance these professional expectations with the personal expectations they place on themselves.

### Challenges (Before)

During the interviews each of the teachers expressed views around the challenges they experienced whilst organising international study tours. Initial discussions centred on the challenges before the study tour around the planning and administration of the study tours with sentiments such as:

There’s so much paperwork that has to be done before I can even advertise a trip, I have to have an itinerary, a costing, risk management (Brooke)

Many of the study participants expressed their frustration when dealing with parents and obtaining paperwork from them, with one teacher saying:

We’ve got parents who decide to leave getting their student’s passport until the very last minute, because kids are turning 18 and they can get a 10-year passport (Claire).

All teachers interviewed expressed concerns over the workload expectations and the time given to them to complete the tasks involved in planning, organising, and leading the tours. Indeed, lack of time was one of the biggest challenges highlighted by the teachers with sentiments such as:

You really need somebody who really wants to devote a lot of time and energy to it, and I mean it’s quite a heavy burden on your shoulders (Helen).

A further issue identified by the participants was the attitude of teacher colleagues and parents towards the study tours, who viewed them as nothing more than a ‘free holiday’. The teachers interviewed all shared that they experienced a struggle for recognition for the time, effort and commitment needed to be involved in leading international study tours, for example:

Some of the parents even say, so are we paying for you then? I say, yes you are because if I was paying for myself, I wouldn’t be taking your kids with me (Edward).

Sometimes there are staff members that will say you’re on another trip—they will head out at 3.30 they’ll get in the car and go home.”… “They don’t see the years of planning that go into the trip (George).

Some teachers commented on the struggles and challenges of fundraising, such as:

Two years to fundraise, and that’s two years of real commitment and hard work (George).

### Challenges (During)

The teachers discussed the challenges involved whilst overseas on the study tours, particularly the obligations and responsibilities that teachers are required to provide adequate supervision for students entrusted by their parents into the school’s care. These responsibilities extended beyond the organisational and logistical aspects of the tour to the safety of students and included both the mental and physical health and wellbeing of the students. A range of teacher’s remarks are indicative of the challenges associated with these responsibilities including:

Half the group were at various stages of illness, and I had to make them all go to the medical centres, it was nearly my last trip. Stress levels up there. I thought I was going to lose my job (Brooke).

We leapt off a boat once, and it was fortunately me that did this; I leapt onto a wharf and the whole wharf collapsed and I went right through it, because it was rotten. That scared me (Claire).

One teacher discussed the distress of losing a student:

So, we lost him in a department store in Berlin—didn’t come to the meeting point…he was sitting under an escalator somewhere reading the newest Harry Potter book, unaware of what was happening. I only found out later that he had Asperger’s Syndrome, and the parents had not declared it (Helen).

The interviews suggest that teachers feel they are in a vulnerable position when escorting and supervising adolescent students on these international study tours. For many of the students, it is their first time away from home and their parents and, therefore, dealing with students’ motions, stresses and anxieties is another area the teachers must be prepared for. Whilst discussing these issues, it emerged that students often suffered from homesickness and loneliness. Other teachers recalled the emotional experiences of dealing with unexpected events, such as sexual-advances and harassment, as well as serious incidents involving death, for example:

We had a car accident one time which was just horrific; came round a corner and here’s this man dead on the road (Andrea).

The cooks in the hotel—well it was the lodge we were staying at, started to make advances to some of our boys (Claire).

They were the three younger, long-haired blondes. By the time I took them out, we’d had three marriage proposals in about two and a half minutes. It was fun and it was good-humoured, but it may not have been; it equally could have been dangerous (Debbie).

Comments such as these, demonstrate the seriousness of the situations that teachers must deal with highlighting the pressure and stress the teachers experience when travelling overseas with a group of adolescents and, the personal challenges involved in taking responsibility for their pastoral care to ensure their physical and emotional wellbeing.

A further challenge mentioned by many teachers pertains to managing the student’s behaviour, especially the consumption of alcohol, for example:

We had to send a couple home, these two had sneaked out of the little accommodation hotel thing we were in, late at night/early in the morning, went to a bar (Frank).

Because the teachers considered that they are held personally responsible for students’ safety and wellbeing, the risk of breaches of this “duty of care” were worrisome and most teachers were of the view that there could be negative repercussions for their professional career should a student be injured, arrested, or be involved in any negative activities.

### Challenges (After)

A further important finding is that many teachers did not experience a sense of relief after one tour was completed but reflected on their feelings that there was a constant cycle of planning and preparation, delivery, and then post-tour that the process of planning and organising for the next tour started immediately.

The challenge of managing the workload associated with the planning, organising and delivery of the international study tours were common. Remarks such as: *“Personally myself I arranged five trips,—I don’t do it every year—it’s so much work and it’s so much to organise—do it every second year.” (Andrea).*

The teachers’ interviews revealed that a diverse range of challenges were experienced because of their roles in these high school international study tours. A commonly held view was that these challenges were not understood by school management, parents, and fellow teachers. In some cases, these people were disparaging the teacher’s involvement and characterized their leadership of the international study tours as a “free holiday”. These derogatory experiences made the teachers question their personal and professional involvement and weigh up these negative experiences against their perceived positive educational value for the students.

### Navigating

All the teachers interviewed expressed views on the strategies they developed and applied to navigate the expectations and challenges that had been placed upon them. Whilst planning the study tours there were differences in the way in which the teachers approached the organisation of the study tours, how they managed their time and workload. Some teachers transitioned to using specialist commercial tour operators to undertake many of the logistical travel planning aspects of the tour. The value of this kind of approach is evident in the following remark:

I’ve had a few people recommend Defining Moments to me. Our other classics teachers use them for classics trips, they just make the planning so easy (Edward).

Despite this trend for some teachers to “outsource” the travel logistics to a private provider, several teachers continued to plan and organise the study tours themselves, for example:

I didn’t want to do it with an organisation because the tour operators are so expensive. So, we used all my private and my school contacts (Helen).

These varying approaches suggest that navigating the cost and affordability and balancing this was a difficult part of organising the study tours. During interviews with the teachers, initial discussions on these issues centred on planning and revealed parent’s low-price expectations, with comments such as:

We have to sell the benefits, inclusions and the value, breaking the cost down (Hilary);

Teachers were also conscious of the additional charges incorporated into the study tours, such as the cost for the teacher’s place, and relief staffing to cover their responsibilities in the school whilst they were away.

A key challenge, identified by all the teachers was the increased workload involved for them and the limited time allowance/compensation (in terms of release from in-school teaching allocations) given to them from their school. Teachers navigated their workload challenges by making choices, to give up their own personal time and to work after school hours. Consequently, teachers were facing a work-family conflict and a deterioration in the quality of their homelife, and time spent with family. Examples of comments which reflected these concerns were:

I’ve got a full teaching load, I’m head of learner support—I find time on my weekends, over the holidays, before school—that’s when I organise the trip (George).

When I went to pick up Jenna from her day care when we got back to New Zealand, it just brought tears to my eyes; this little girl that finally could see her mother again after 21 days (Debbie).

Teachers were confronted with a wide range of personal dilemmas when organising and participating in the study tours, this involved implementing a variety of strategies to navigate and balance their workload and family commitments.

### Negotiating

The data revealed that all the teachers had strategies in place to navigate and negotiate student behaviour. Before the study tours, student selection was based on the positive behaviour of the student in the school environment, enthusiasm for the subject, class attendance, attitude, relationship with teacher, etc. Student behaviour was generally discussed and evaluated through a collaborative process usually involving the staff arranging the international study tours. For several schools this involved a hierarchical selection process starting with the subject teacher, Head of School or Department, Principal and often included student counsellors.

Most teachers knew the students on a personal level before selecting them for participation in the international study tour. For example, Debbie stated:

As a secondary school teacher, you kind of get a pretty clear idea about the ones that have got their head screwed on, and the ones that haven’t quite done that yet.

Through this familiarity, awareness and understanding of the character of the students the teachers were able to make informed judgements and decisions around the student selection process to ensure the suitability of the students for the international study tours.

To manage student behaviour during the international study tours, each of the teachers spoke of “behaviour contracts”, as a means of setting expectations, negotiating, and controlling student behaviour. These behavioural contracts were put in place to safeguard the school’s reputation, and to protect the teacher in charge, since the teachers were taking on responsibility for the welfare and wellbeing of the student group whilst travelling.

Teachers identified that a further important influence on student selection was affordability. The cost of the international study tours ruled out many students from participating in the international study tours and many teachers lamented the lost opportunity for these students. For example, one teacher recalls a student:

Who didn’t get to go on a classics trip, and she was one of those students that, if I had money, I would have taken her, because she was so—she loved it so much, and it was one of the kinds of really hard moments (Edward).

Thus, the financial status of student’s families was an important determinant on student’s ability to participate in the tour and many teachers struggled with the ethical dilemma of inequality of opportunity for students.

## Discussion

The consensus that study tours are beneficial is widely reported (e.g. Lagos et al., 2019; Penning et al., 2019). These benefits are heavily promoted to both parents and students by schools, teachers and, increasingly by private travel businesses who partner with schools in selling these tours (Schänzel & O’Donnell, 2021). Lewin (2010) points out that international study tours can be seen as a form of consumerism and marketing masquerading as academic experiences. However, this study gives important insights into the motivations, influences and experiences of high school teachers organising international study tours.

**Fig. 3:**
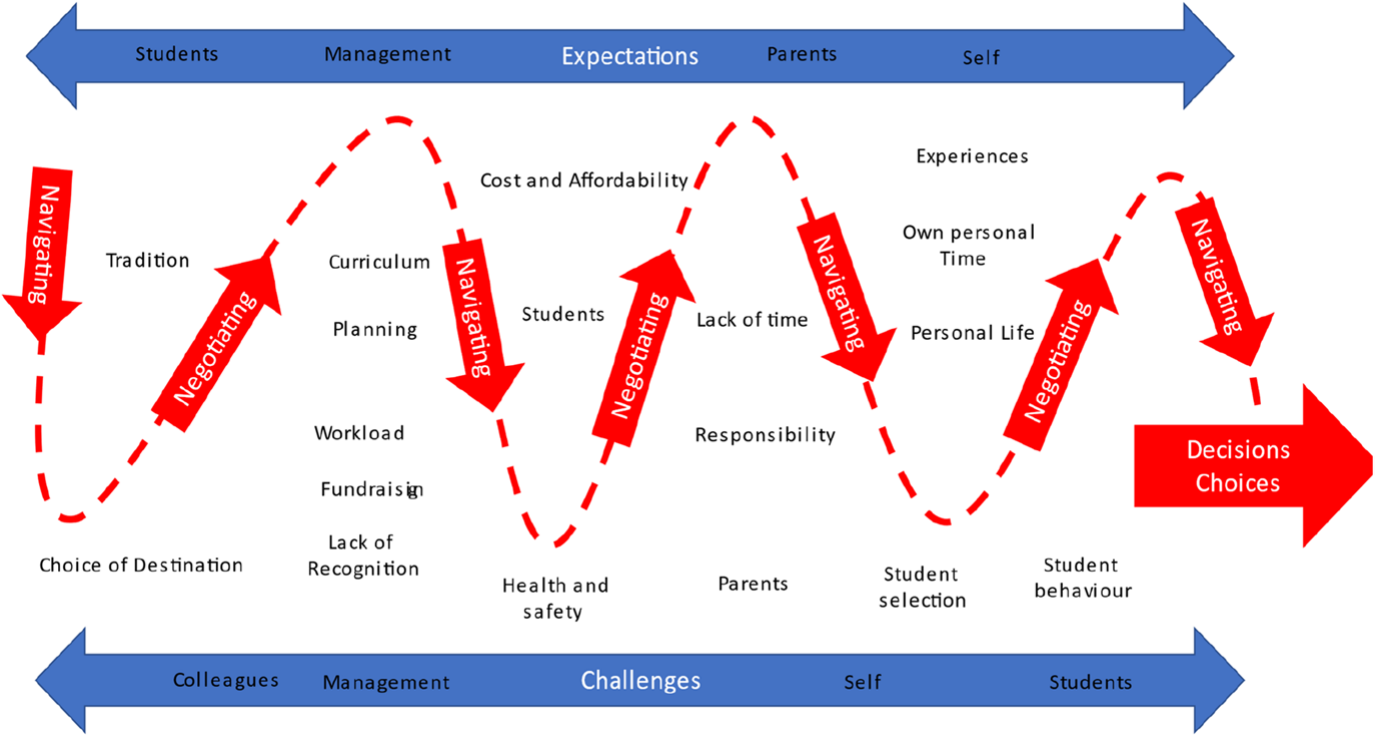
Navigating and Negotiating Learning Experiences Theory

From these data a constructivist grounded theory entitled “Navigating and Negotiating Learning Experiences” is proposed. This theoretical model depicts the range of decisions and choices made by teaching staff when planning, organising, and leading the international study tours. The theory’s main categories are graphically represented in Fig. 3. The arrows depicted in the model demonstrate the temporal pathway the teachers’ experiences followed as they progressed in the organisation of the study tours as they navigated and negotiated their way through the expectations and challenges placed upon them by school management at an institutional level, by parents, by students, and from themselves. The theory demonstrates how all the teachers had strategies in place to navigate and negotiate the expectations and challenges they experienced and how personal motivation influenced their choice to remain in their roles as leaders of these study tours.

The teachers within this study felt a sense that both high school students and their parents had a general expectation that their school would organise a variety of international study tours each year. These expectations were reinforced by expectations from the school management that these tours were a standard aspect of the school’s educational offerings for their students and often used as educational products to enhance the school’s reputation, inferring that the cultural and social capital tied to particular schools contributes to the success of the organisation. Whilst this study did not seek to address the motivation of school management, it is an area that warrants further investigation. Since, teachers in subject areas where these tours had been a standard feature felt considerable pressure to meet these expectations to plan, organise and lead these tours even though such work did not form a formal part of their employment agreement with their school.

Prior to departing on the study tour the teachers involved often experienced an increase in their individual workloads based around the specific expectations and responsibilities for organising and planning the study tours, fundraising, student learning, and health and safety requirements. Teachers workloads have been the subject to a range of studies concluding that teachers have become overloaded with work expectations which is contributing to burnout and teachers choosing to leave the profession (e.g., Easthorpe & Easthorpe, 2000; Swaim & Swaim, 1999; Timms et al., 2007). This over-work is often identified as a result of additional administration, paperwork, and the pastoral care of students as opposed to the actual in-class teaching of students (Timms et al., 2007). This research suggests that whilst teachers are generally satisfied in their educational roles, it is these additional duties that are stressful. Many teachers navigated the workload challenges by giving up their own time and taking work home with them (Easthorpe & Easthorpe, 2000).

This study confirmed that organising international study tours required a great deal of personal commitment with teachers needing to work additional time outside of their school hours. The findings emphasised a tension between the expectations placed on the teachers (by the school, parents, and students) and the level of personal time commitment required. Teachers universally shared that they experienced a struggle for recognition for the extra time and effort they put into planning and organising the study tours. The findings emphasise that the offering of these international study tours relies on the resilience, commitment, and passion teachers have for these roles and their willingness to give up their own personal time to ensure the tours can happen.

Teachers in this study were expected to make informed choices and critical decisions regarding the international study tours but often found these responsibilities stressful, as decisions were governed by the Ministry of Education guidelines, the Board of Trustees, student’s and parent’s expectations, and other colleagues’ perspectives when organising the study tours. These decisions were made even more complex because the teachers had to consider the selection of appropriate destinations and experiences that supported and empowered students to learn; health and safety management issues, fund-raising and promotion of the tours.

The findings showed that teachers used logical decision-making strategies drawing on previous experience of organising study tours to specific destinations to influence the choice of future destinations and experiences. These findings resonate with Pearce and Lee (2005) and Karl et al. (2020) who suggest that past travel experiences influence the types of perceived risk associated with international *travel*. Teachers identified further challenges around the stringent health and safety requirements and additional administration prior to departure and, that their obligations and responsibilities to ensure student’s safety was a major focus during the study tours. The data from this study provides evidence that the teachers were highly aware of the health and safety requirements and personal risks associated with taking students on a study tour to international destinations and that this was a source of concern for them. This is not surprising because there have been a number of tragic incidents reported in the New Zealand media with students and teachers losing their lives on overseas study tours (e.g., Garside, 2013; Leask, 2013). Hence, health and safety requirements often influenced the planning and organisation of the study tours by determining the destination, opportunities, experiences, and attractions offered to the students.

It is evident from this study that teachers had concerns over monitoring and managing student behaviour, especially the consumption of alcohol. These findings are consistent with Carr (2011) and Horner and Swarbrooke (2020) who concluded that alcohol consumption on school trips is common in adolescence. Moreover, teachers were expected to deal with a wide range of student emotions ranging from feelings of excitement, apprehension, happiness, anxiety, loneliness/homesickness, and sexual harassment. Although, these emotional responses have been commonly reported in the literature (e.g., Hains-Wesson & Ji, 2020; Vogel, 2018), ensuring the wellbeing and safety of students can place teachers in a vulnerable position, causing personal pressure and emotional strain on the teaching staff involved as they feel responsible for the welfare of the students and their school’s reputation. This raises the question of whether teachers should personally shoulder the responsibility of taking students on overseas trips as it is emotionally demanding. Dudley-Marling and Michaels (2015) and Kell (2018) suggest that expectations placed on teachers to assume this responsibility stem from society with parents making judgments about educational establishments and teaching practices in relation to student success. This study substantiates these claims, as the expectations from the school community place additional pressures on teachers to continually organise international study tours. Consequently, international study tours have become embedded into the school culture and are often used to enhance the school’s reputation.

The teachers in this study had strategies in place to navigate and negotiate the challenges they experienced. For example, tour operators were utilized to organise packages as a time saving measure. These findings are consistent with those from Roberts et al. (2018) who found that tourism teachers adopted the same approach. Whilst this approach saved teachers time, there are concerns that this allows travel organisations to establish partnerships with schools as trusted and reputable providers of educational travel allowing consumerism to infiltrate educational spaces (Hogan & Thompson, 2020). Another strategy utilized by many of the teachers to navigate student behaviour was acting as gate keepers in the selection process. This finding corroborates the ideas of Sanz and Morales-Font (2018) who noted students were selected on the basis of both academic results and individual interviews. In this sense, international study tours can be seen as a form of distinction. Affordability was also a determining factor in the selection process, highlighting disparities between students even within higher decile 10 schools. Furthermore, the lack of lower decile schools within this study demonstrates there are significant inequalities in the opportunities available to students, emphasising that perhaps study tours are not curriculum-oriented but cultural, social, and economic capital based with distinct links to educational consumerism. It is evident from the data that affordability caused several challenges, with teachers aware of the criticism around the high costs of the study tours, the challenges to manage parent’s price expectations, and disparities/inequity in the inclusion of students in these experiences. The findings support criticism that the high costs excluded some students from participating in study tours and that teachers were conscious of the ethical dilemma and equity issues of these educational experiences not being available to all. The findings provide additional evidence to support those reported in other studies where debates around high cost, affordability and barriers to education have been prevalent (e.g., Bos et al., 2015; Gausden, 2018).

This study suggests that the teachers faced several personal and professional dilemmas and were often subject to pressures and challenges at an institutional level which placed demands on their own personal lives. This is a point that has generally been overlooked in previous research with the focus being predominantly on the benefits to students and their learning (Campbell-Price, 2014; McGladdery & Lubbe, 2017). Given the significance of the teacher’s role in organising international study tours, this is short sighted. While there is maybe some overlap between the personal benefits to teachers and the benefits to teaching, it is worth noting that the findings from this study did not show that teachers strongly identified these benefits. For example, no teachers mentioned that their roles in organising the study tours was helpful for their career advancement (through promotion). Furthermore, no teachers reflected on their own personal learning and growth because of their roles. What teachers focused on were the challenges their roles created for them and the sacrifices they made. Another strong and commonly held view was the motivation to organise the study tours was driven by their personal passion for teaching and learning and the sense of value of these experiences for the student’s education and growth.

Therefore, despite the challenges and sacrifices experienced by these teachers and the many personal and professional responsibilities, the findings suggest a key motivator was job satisfaction and replicating their memories of their own experiences as adolescents traveling on school trips. Since the role of teachers in organising and leading international study tours is a critical one, these individual experiences have formed the basis of the navigating and negotiating learning experiences theory offering important insights into their role.

## Conclusions

The findings from this study, focused on New Zealand high school teachers who organise international study tours, reveal that these educational tours are a massive personal undertaking for the teachers involved. The teachers identified that the expectations, pressures, and challenges placed upon them from the school community and parents had implications for both their personal and professional lives. Findings also showed that while the organising and leading of these tours were stressful and emotionally demanding they were also rewarding and personally satisfying for the teaching staff involved.

This study has several important implications for the education sector. It is clear from the teachers’ comments that on both a professional and personal level they are committed to their role and wish to live up to the expectations placed upon them, however they require more support at an institutional level. Findings show that while management in high schools were generally supportive of international study tours led by teachers, they were less supportive regarding the increased workloads and responsibilities placed on the teachers involved. Additionally, teachers leading these tours were of the view that most of the students participating and their parents were unappreciative of the extra personal time and effort teachers put into organising and leading these study tours to ensure their success. Hence, teachers struggled for recognition for the time and effort they put into organising the study tours and questioned whether the sacrifices involved were worth it. This research suggests, therefore, that there is a disparity between the importance of the study tours for high schools and their students and the formal recognition of the role teachers play to ensure their success.

This study provides new theoretical insights into this growing component of educational tourism by introducing the navigating and negotiating learning experiences theory. The focus on exploring the teacher’s perspective as key brokers and leaders of these experiences has not been reported before. In the New Zealand context, government educational agencies, school boards and principals all encourage the expansion of education outside of the classroom. Furthermore, the growth in opportunities for high school age students to travel internationally and to engage in learning as part of an organised tour are expanding. In many cases they are promoted as an important benefit for students attending a specific high school and are expected as a regular offering for students. The role of teachers and the pressures placed on them as the key facilitators of these experiences have not been formally recognized and the teachers involved received no specific training or explicit support or recognition in their workloads.

The scenario exposed by this study reveals potential risks. Teachers bear responsibility for the safety of young adolescents when travelling internationally on these tours. Parents and school management place these expectations on teachers and the “in loco parentis” legal and moral implications for teachers to serve as guardians of these children is a role that teachers are not specifically trained for. The teachers themselves accept these responsibilities and feel the stress associated with the role. The risk of “burn-out” as teachers become fatigued and tired of the work, responsibility, and stress is an important implication of the findings of this study.

The Navigating and Negotiating Learning Experiences Model derived from this research is a valuable schematic which theorizes the perspectives and tensions involved in teacher’s experiences in the high school international study tours. The study was limited in participation with no low decile schools represented. Further research which seeks to apply and test this model in other contexts, including lower decile schools to develop national insights and the effects of a global pandemic, is worthwhile. Such additional investigation can further add to our limited understanding of this important aspect of educational tourism experiences. With restrictions on travel now lifted in New Zealand, post-pandemic it is expected that international study tours will resume and continue to grow with many schools encouraging students to travel overseas.
